# Tourists’ Safety Perception Clues in the Urban Forest Environment: Visual Quality, Facility Completeness, Accessibility—A Case Study of Urban Forests in Fuzhou, China

**DOI:** 10.3390/ijerph19031293

**Published:** 2022-01-24

**Authors:** Hongda Wang, Jing Ye, Muhammad Waqqas Khan Tarin, Yueyan Liu, Yushan Zheng

**Affiliations:** College of Landscape Architecture, Fujian Agriculture and Forestry University, Fuzhou 350002, China; 1191775034@fafu.edu.cn (H.W.); Jingye@fafu.edu.cn (J.Y.); waqas_tarin@yahoo.com (M.W.K.T.); 1201775028@fafu.edu.cn (Y.L.)

**Keywords:** urban forest, safety perception scale, structural equation model

## Abstract

The service quality and safety perception of urban forests are important factors that influence tourists to choose them as recreation destinations. This study aims to propose a theoretical model of multivariate relationships to explore the relationship between service quality (including visual quality, facility completeness, and accessibility) and safety perception to examine whether visual quality, facility completeness, and accessibility on tourists’ safety perception in the urban forest and to explain the specific reasons for the impact. We collected sample data from many urban forest green spaces in Fuzhou through a two-stage field survey (N = 891), and controlling for potential confounders, a structural equation model was used to estimate relationships. Safety perception was divided into safety environment perception, control perception, and safety emotion. Visual quality of an urban forest positively affected safety emotion. Traffic accessibility positively affected control perception. Facility completeness had a positive impact on safety emotion and control perception. Both safety emotion and control perception played an important intermediary role in improving the perception of a safe environment in the multivariate model. Visual quality, facility completeness, and accessibility all had a positive impact on tourists’ safety perception of urban forests. The findings suggest that improving the service quality of a green space can effectively improve tourists’ evaluation of the safety of the urban forest environment. Specifically, tourists’ psychological tolerance to threats and their self-confidence in survival can be enhanced by improving the service quality of a green space.

## 1. Introduction

An urban forest refers to the natural forest area within or near a city [1]. Their geographical advantages and natural environments make them attractive places for recreation. A forest has a unique aesthetic value compared to a park. Forest bathing, as a kind of natural therapy, has been shown to promote physical and mental health [2]. This activity is persuading more and more visitors to undertake forest recreation and promoting the development of forest tourism in regional economic growth. Further, compared to landscape parks, forests, as a wilder natural area, are often considered to be more dangerous. For this reason, some tourists restrict their travel choices, and few select forest environments, especially for outdoor parent–child activities [3]. Therefore, it is particularly important to eliminate tourists’ worries and prejudices about the urban forest environment.

To make the forest a popular recreational green space for tourists, it is essential to balance the perceived conflict between the recreational appeal and the threat of wild nature. Improving tourists’ recreation experience while convincing tourists that these environments are safe has become a key research issue in urban forest recreation development. Visitors enjoy recreation in the planned activity area in urban forest green space. Therefore, improving the service quality in the activity space can enable tourists to have a better recreational experience. According to the broken window theory [4], a well-managed and orderly green space can make tourists feel safe. Improving the management of an urban forest can be achieved by enhancing the green space’s social attribute, which is the convenience of recreational activities in the environment [5]. The service quality that reflects this trait may affect tourists’ safety perception in the urban forest. To test this hypothesis, it is necessary to have a deeper understanding of the internal relationship between safety perception and urban forest service quality.

Perception can be defined as the experience process obtained through mental understanding and processing after acquiring external information through the senses [5]. It is collectively influenced by external environmental information, emotion, and social factors. This theoretical use is also used to explain people’s perception of safety in the environment. Safety perception is a process by which people selectively filter information related to safety in the environment and pre-judge whether they are in a safe state. Factors such as objective environment, instinct, individual experience, knowledge, and social propaganda can all influence people’s recognition of safety clues in the environment [6]. Fisher and Nasar [7] proposed in their study of campus crime that in addition to prospect and shelter environmental characteristics, the assessment of escape possibility in the environment is also an important factor affecting safety assessment. Leon Van Rijswijk [8] further summarized the factors influencing safety assessment into three parts: the predictability of the environment, individuals’ behavioral control over potentially dangerous situations, and the influence of personal anxiety traits on environmental safety judgment. Although previous studies on environmental safety assessment have included some theories on safety perception, few studies have summarized them. The safety assessment of built parks tends to be divided into personal, social, and physical environments from the macro-level perspective [9]. However, people’s forest environmental safety is strongly correlated with concerns about personal safety, which is also a focus in the literature of safety perception. From the perspective of self-assessment, our research selects three main bases for personal safety judgments, environment, control, and emotion, to collect and organize the literature.

Based on the existing theoretical research results, we further clarify the three dimensions of personal safety perception and put forward the theoretical concepts of each dimension, which are the main theoretical basis for the subsequent compilation of the safety perception scale. We named these three dimensions safety environment perception, control perception, and safety emotion.

Safety environment perception is a predictive judgment that the environment is non-threatening. From the perspective of space and time, the prediction of environmental safety needs to include the judgment of both the certainty and uncertainty of environmental conditions [10]. The basic condition to judge the safety of an environment is to ensure that the space is not suitable for concealment and entrapment [11]. Concealment refers to criminals or dangerous people hiding in the space. A space with open views and no conditions for concealment is considered to be safe. Entrapment occurs in a space in which escape is obstructed. Even though there is no hazard in the environment, a physical obstacle on a path makes people feel unsafe. The uncertainty of a safe environment comes from potential threats that cannot be immediately observed when entering the environment and are assumed based on people’s own experience [9]. The forest environment is different from parks, as in addition to the uncivilized behavior associated with crime, such as empty or dilapidated infrastructure construction, wandering youth, and homeless people [12,13], there are more potential threats from the wild environment itself, including dangerous animals and insects, deep water, extreme weather, getting lost, etc. [14].

Control perception is an assessment of behavior feasibility that ensures one’s safety under an imaginary dangerous situation, which can enhance one’s confidence in safety [15]. In the natural environment, risk management is very important. Regardless of whether the main body controlling safety is oneself or others, it is an active preparation against potential dangers [16]. Approaching positive stimuli and avoiding negative stimuli are people’s instinctive behavioral tendencies [17] and are basic strategies for individuals to deal with threats. Therefore, the most basic behavioral tendencies of self-control perception can be divided into two types: approaching shelters and staying away from danger sources. The control perception from others can be regarded as a positive feedback of the “eyes on the street” effect. Individuals’ judgment on the possibility of assistance from others in the natural environment is mainly based on two points: informal social control carried out by other green space users and supervision by green space managers [18,19]. The natural control of many users over the green space makes them more likely to notice abnormal behaviors or dangers and provide help or call the management of the green space for rescue, which is also a benefit brought about by sufficient user visits [20]. Therefore, control perception can be divided into four aspects: escape, shelter, help, and rescue.

Emotional factors are rarely mentioned in studies on safety perception, but many studies have confirmed that negative emotions can greatly affect the perceived results [21,22]. People’s negative emotions towards safety are mainly expressed as anxiety and fear, which make them psychologically reject the strange natural environment [23]. In addition, loneliness is also an important factor affecting people’s safety perception in the forest environment; companionship can effectively solve this problem, even if the companionship is only in the form of a human voice [24,25]. Positive emotions are rarely mentioned in studies on safety perception, which may be because, as a basic survival need, safety does not cause rich psychological and emotional changes. The neutral position of safety psychology is often determined by negating negative emotions. However, some studies have proposed that positive emotions can also affect safety perception. As a positive emotional state, comfort can improve people’s affinity to the natural environment and inhibit the tendency to perceive threats [26].

Visual quality, accessibility, and facility completeness were selected as specific indicators of the quality of urban forest recreation services in this study. The recreation service of an urban forest closely relates to the needs of tourists. As one of the most basic needs of people, safety is the focus of research on recreation services. In several nature recreational studies, accessibility, facility completeness, and visual quality have been proven to be needs that tourists expect to be met, as well as external manifestations of the management level of natural green space [27,28,29]. Visual quality is the charm of natural green space and viewing angle presented to tourists after aesthetic selection. It fulfills the needs of tourists to get close to nature. Accessibility refers to the convenience of transportation from the urban residential area to the urban forest recreation area and reflects the closeness of service connection between nature and society. Facility completeness reflects the completeness of facilities in the man-made environment of an urban forest and is the basic guarantee for tourists to conduct social activities and outdoor sports. Facilities, transportation, and landscape are the most frequently mentioned environmental elements in environmental safety assessment research [3,30]. Existing research results have proven that the lighting quality of space can affect the perceived safety of the environment at night and further influence people’s route choices [6]. The planning and designing of public spaces such as parks with existing facilities like public telephones, fences, landscaping, and monitoring are believed to reduce the incidence of crimes [13]. In urban natural areas, the optimization of vegetation features, such as by vegetation density adjustment and vegetation space design, has been proven to have a positive impact on personal safety perception [31], which can reduce the degree of threat from wild vegetation. Maintenance is one of the important principles of crime prevention through environmental design (CPTED) theory. The maintenance trace of vegetation can indicate that the environment is under supervision and protection and thus improve tourists’ evaluation of environmental safety [32]. At the same time, the accessibility, scale, naturalness, and other qualities of urban natural areas are also believed to affect the social and psychological benefits perceived by tourists in the natural environment [33]. These research results show that there is a connection between visual quality, accessibility, and facility completeness and safety perceptions, but few studies have clarified the specific relationship between these three service qualities and safety perceptions and explained why these three services can affect tourists’ assessment of environmental safety.

In this empirical study, our main research purpose is to test the hypothesis that facility completeness, accessibility, and visual quality can affect tourists’ perception of safety in urban forests. To have a deeper understanding of the relationship between the three service qualities of urban forests and safety perception, this study is divided into two stages. In the first stage, we divide the internal dimensions of safety perception, which will help to obtain more comprehensive and specific safety perception assessment results. Based on collating and summarizing the existing research results, we construct a theoretical framework of safety perception and conduct field application tests to verify the scientific nature of the division of the internal dimensions of safety perception. In the second phase, we explore the impact of facility completeness, accessibility, and visual quality on the three safety perception dimensions. In the specific research process, we also explore specific paths through which three service quality measures affect environmental safety assessment and build the internal mechanism model between them. This helps us to have a deeper understanding of the internal reasons why these three service qualities affect safety perception and analyze the differences and commonalities between them. This can provide a reference for formulating effective measures to improve the perception of urban forest safety, and also has important enlightening significance for the design and management of urban forests.

## 2. Materials and Methods

### 2.1. Overview of the Study Area

**Figure 1:**
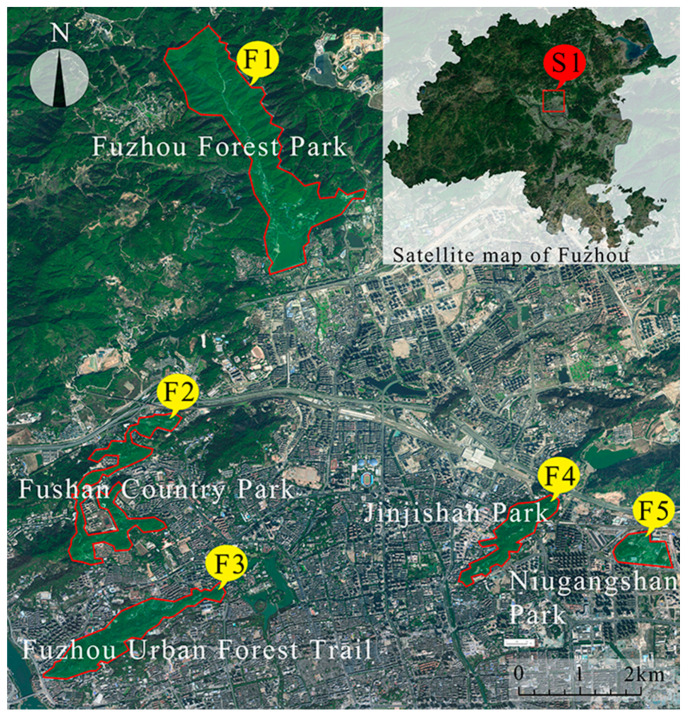
Locations of 5 Urban Forest Recreation Areas in the main city of Fuzhou: S1, the location of the main city of Fuzhou; F1, Fuzhou Forest Park; F2, Fushan Country Park; F3, Fuzhou Urban Forest Trail; F4, Jinjishan Park; F5, Niugangshan Park.

The research was carried out in Fuzhou from 15 September 2020 to 16 April 2021. Fuzhou, as the capital of Fujian Province, is located on the coast of the East China Sea. The landscape of Fuzhou is a typical estuary basin surrounded by mountains. The altitude of its mountains is mostly between 600~1000 m, and it has rich forest resources. In the first stage of the study, five urban forest recreational lands in Fuzhou were selected to verify the safety perception scale (Figure 1): Jinjishan Park, Fuzhou Urban Forest Trail, Fuzhou Forest Park, Fushan Country Park, and Niugangshan Park. These are the five green spaces with the highest proportion of natural forests around the main city of Fuzhou, including two geographical elements of an urban interior and urban edge. Fuzhou Forest Park (F1) is a comprehensive park that integrates scientific research and tourism. Fushan Country Park (F2) has effectively highlighted the beauty of the wilderness landscape through planning and transformation. Fuzhou Urban Forest Trail (F3) is completely built in the forest, retaining the original appearance of the natural mountains and forests. Jinjishan Park (F4) is located at the foot of Jinji Mountain and has many scenic spots and historical sites. Niugangshan Park (F5) is a small woodland park that is easy to explore.

To further study the relationship between urban forest service quality and safety perception, Fuzhou Urban Forest Trail was selected to be representative of an urban forest green space in the second stage, to reduce the influence of other interference factors on urban forest service quality and safety perception. Fuzhou Urban Forest Trail is located in the city’s Gulou district. The main part of the trail is built along the ridge line of Jinniu Mountain. The total length of its axis is 6.3 km, and the total length of the loop is about 19 km. It is the first urban forest walkway in China to adopt a steel frame hollow-out design. All along the trail is a natural forest landscape with a unique canopy and a wide variety of species, providing a natural and novel experience for visitors. Fuzhou Urban Forest Trail has now become the first choice for forest strolling in Fuzhou. It has a relatively large number of tourists, which makes it a feasible and convenient location for this study.

### 2.2. Methods of Research Phase 1

The internal dimensions of security perception are mainly divided based on the results of previous studies, so a structural validity test was required. This was the prerequisite for the follow-up research. To obtain information about tourists’ safety perception quickly and effectively, it was important to develop a set of feasible safety perception measurement tools. This was also an important way to test the rationality of the safety perception dimension division. 

#### 2.2.1. Questionnaire

The theoretical framework of personal safety perception that we collated compiles four measurement items for each of the three dimensions of safety perception. To make the project description simpler and more user-friendly, without jargon, we referred to some topics and item description methods of Han’s self-rating restoration scale (SRRS) and state-trait anxiety inventory (STAI) [34,35]. After a collective discussion between the questionnaire designers, a list of scale items (12 items) was formed. Later, the researcher conducted interviews at the research site to ensure the reliability of the questionnaire. The researcher compiled the safety perception scale (SPS) after making a semantic adjustment and supplementary annotation to the items’ descriptions that were difficult to understand or were ambiguous and then conducted a preliminary survey. In addition to the 5-point semantic difference scale used in the measurement of emotional description, a 5-point Likert scale was used for the other items. The final questionnaire was formed after further adjustment according to the results of the preliminary survey.

#### 2.2.2. Participants and Procedure

The study was conducted from 15 September to 20 October in 2020. To ensure the scientific nature of the dimension division of the safety perception scale, the collected test data were provided by users of several urban forests and green spaces in Fuzhou according to their experience, reducing the interference of the environment on safety perception. The questionnaires were randomly given out in five urban green forest areas during legal holidays, weekends, and working days. The items in the questionnaire were randomly sorted. Before the survey, the purpose of the study and the matters needing attention were explained in detail to those who were willing to complete the questionnaire to improve the efficiency of questionnaire collection. Ninety questionnaires were sent out in each of the five urban forest green spaces. In total, 450 questionnaires were sent out, and 411 valid questionnaires were collected, for an effective questionnaire rate of 91.3%. Among the respondents, 187 were male and 224 were female; 17 participants were under 18 years old; 177 people were between 18 and 25 years old; 112 people were between 26 and 35 years old; 56 people were between 36 and 45 years old; 32 people were between 46 and 55 years old; and 13 people were 56 years old and above. 

### 2.3. Methods of the Research Phase 2

**Figure 2:**
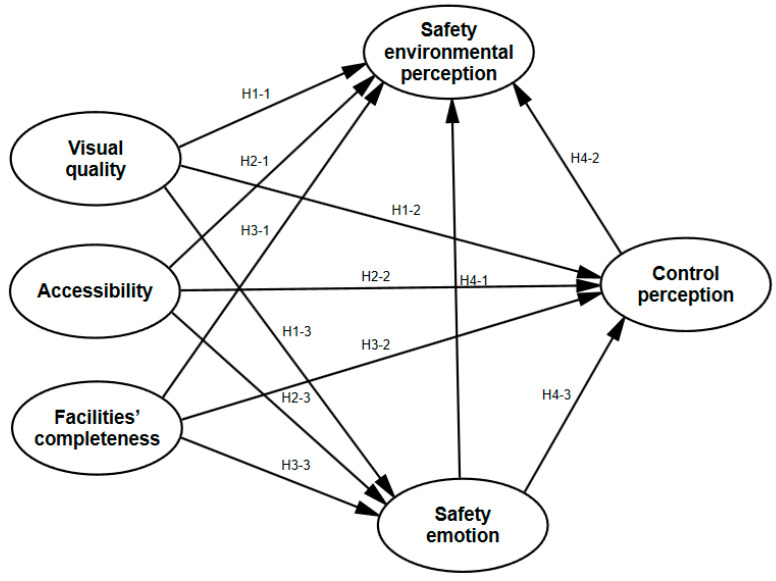
Structural equation modeling (SEM) diagram.

Based on the research results of phase 1, we proposed the basic theoretical conception of safety perception. In phase 2, we further verified this theory and applied it to research on the relationship between service quality and safety perception. The intrinsic process of safety perception can be understood from the perspective of human–environment interaction. According to the human environmental interaction (HEI) model [36], human feelings towards social and physical environments can be influenced by individual reaction tendencies. Control perception can be seen as a person’s tendency to react to threats and as an intermediary variable between safety emotions and safety environment perception. The purpose of phase 2 was to explore the impact of facility completeness, accessibility, and visual quality on environmental safety assessment. To achieve the purpose of this study, we proposed the structural equation model (SEM) relationship between variables as shown in Figure 2 and made the following hypotheses.

**Hypothesis** **1** **(H1):** The three internal dimensions of safety perception are all affected by the visual quality of the landscape.

**Hypothesis** **1-1** **(H1-1):** Visual quality has a significantly positive effect on safety environment perception.

**Hypothesis** **1-2** **(H1-2):** Visual quality has a significantly positive effect on control perception.

**Hypothesis** **1-3** **(H1-3):** Visual quality has a significantly positive effect on safety emotion.

**Hypothesis** **2** **(H2):** The three internal dimensions of safety perception are all affected by accessibility.

**Hypothesis** **2-1** **(H2-1):** Accessibility has a significantly positive effect on safety environment perception.

**Hypothesis** **2-2** **(H2-2):** Accessibility has a significantly positive effect on control perception.

**Hypothesis** **2-3** **(H2-3):** Accessibility has a significantly positive effect on safety emotion.

**Hypothesis** **3** **(H3):** The three internal dimensions of safety perception are all affected by facility completeness.

**Hypothesis** **3-1** **(H3-1):** Facility completeness has a significantly positive effect on safety environment perception.

**Hypothesis** **3-2** **(H3-2):** Facility completeness has a significantly positive effect on control perception.

**Hypothesis** **3-3** **(H3-3):** Facility completeness has a significantly positive effect on safety emotion.

**Hypothesis** **4-1** **(H4-1):** Safety emotion has a significantly positive effect on safety environment perception.

**Hypothesis** **4-2** **(H4-2):** Control perception has a significantly positive impact on safety environment perception.

**Hypothesis** **4-3** **(H4-3):** Safety emotion has a significantly positive influence on control perception.

#### 2.3.1. Questionnaire

In phase 2, the questionnaire was divided into two parts: service quality and safety perception. In the study on the service quality of the Fuzhou Urban Forest Trail, some researchers have constructed evaluation indices for landscape visual quality, facility completeness, and accessibility [37,38], which have been verified to be effective in the field investigations. The service quality survey in the questionnaire was modified and compiled according to the existing research results, and the scoring standard was also a 5-point Likert scale. The scale and scoring criteria in phase 1 were used to investigate safety perception.

#### 2.3.2. Participants and Procedure

**Figure 3:**
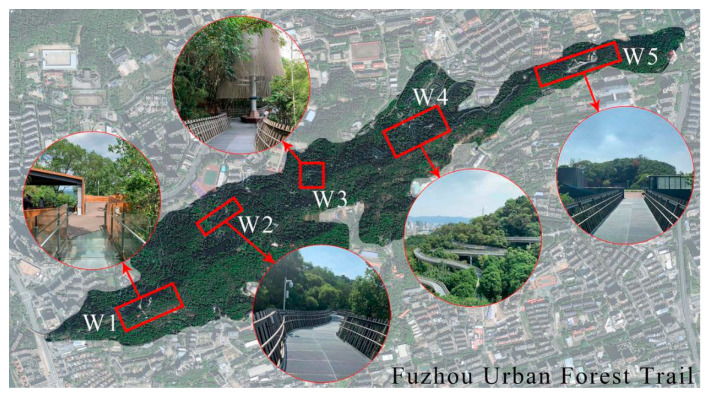
Location of five walking tour areas in Fuzhou Urban Forest Trail. (W1~W5, walking tour area numbers).

This research was conducted from 2 November 2020 to 28 November 2020 and from 20 March 2021 to 16 April 2021, and included working days with few tourists and days off on which more tourists visited the area. One hundred volunteers were invited to participate in a walking tour of a specific distance at five regions with different landscape characteristics on the Fuzhou Urban Forest Trail (Figure 3, Table 1). They later completed the questionnaire about the service quality and safety perception of the forest trail after they were familiar with the surrounding environment. In total, 500 people were surveyed and 480 questionnaires were collected, for an effective questionnaire rate of 96%. Of the participants, 233 were males and 247 were females; there were 33 people under 18 years old; 123 people between 18 and 25 years old; 158 people between 26 and 35 years old; 92 people between 36 and 45 years old; 51 people between 46 and 55 years old; and 23 people 56 years old and above.

### 2.4. Statistical Analysis

SPSS 24.0 and AMOS 24.0 were used for data processing and statistical analysis. This study followed the basic procedures and principles of scale development when designing the questionnaire. Construct validity was mainly embodied by convergent validity and discriminant validity. The Cronbach’s α coefficient of the questionnaire was greater than 0.7, indicating that the measurement has good stability. The SEM was evaluated according to the theoretical perspective and according to the fit quality criteria. The maximum likelihood method was used to estimate the parameters of the structural model. The bias-corrected percentile bootstrap method was used to test the mediating effect.

## 3. Results

### 3.1. Reliability and Validity Test of the Safety Perception Scale

#### 3.1.1. Project Analysis and Reliability Analysis

The results showed that the critical ratio (CR) values of all items were between 18.965 and 28.039, or greater than the minimum reference value of 3.000, and the significance test probability *p*-value was less than 0.001. The score differences of all items were statistically significant. The Cronbach’s alpha coefficient of the safety perception scale was 0.911; this coefficient did not increase when any question was removed, indicating that the scale had good internal reliability.

#### 3.1.2. Exploratory Factor Analysis and Testing

The Kaiser–Meyer–Olkin (KMO) measure of sampling adequacy was 0.907 for the safety perception scale, the approximate chi-square distribution of Bartlett’s sphericity test was 3043.224, and the significance probability value was *p* < 0.001, reaching the significant level. The above findings represent that the sample data were suitable for factor analysis. Based on principal component analysis of the data sample (Table 2), the cumulative variance contribution rate of the three common factors of the safety perception scale was 73.909%. The percentage of the explanatory variance of factor 1 was 25.227%, that of factor 2 was 24.912% and that of factor 3 was 23.769%. The classification results of the principal component factors were consistent with the envisaged theoretical framework and expectations. The three factors were respectively named control perception, safety emotion, and safety environment perception.

### 3.2. Structural Equation Model Testing

#### 3.2.1. Confirmatory Factor Analysis and Test

Among the overall fit indices, the chi-square degree of freedom ratio (CMIN/DF) was 2.660. The root means square error of the approximation (RMSEA) was 0.059, which is less than the reference value of 0.08. The goodness-of-fit index (GFI) was 0.903, the normed fit index (NFI) was 0.910, the incremental fit index (IFI) was 0.942, the Tucker–Lewis index (TLI) was 0.931, and the comparative fit index (CFI) was 0.942; all of these figures were greater than the reference value of 0.9. The results showed that the overall model fits well. The phase 2 study questionnaire used Cronbach’s alpha to test the internal reliability of each category (Table 3). The results were all greater than the reference value of 0.7, indicating that the questionnaire has good reliability.

Construct reliability (CR) and average variance extracted (AVE) were used to detect the testing variables’ convergent validity (Table 4). The results showed that the various factors in the questionnaire of AVE values were greater than the reference value of 0.5, and CR values were greater than the reference value of 0.7. All observed variables had good internal consistency and good convergence in each latent variable.

Discriminant validity of the test results showed that all six latent variables had a significant correlation with each other (*p* < 0.001, Table 5). The correlation coefficients were less than the square root of AVE, suggesting that all latent variables had both a certain correlation and differentiation between each other.

#### 3.2.2. Structural Model

**Figure 4:**
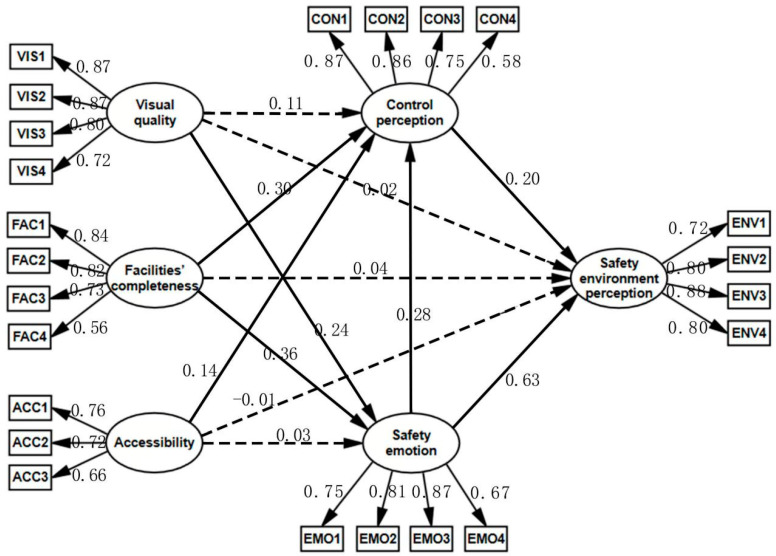
Structural equation modelling results. (Dotted line, hypothesis rejected; solid line, hypothesis accepted).

SEM was used to test the hypothesis of the relationship between service quality and safety perception (Table 6). The best fitting model was shown in Figure 4. The fitting index of the model was CMIN/DF = 2.447, RMSEA = 0.055, GFI = 0.912, NFI = 0.918, IFI = 0.950, TLI = 0.940, and CFI = 0.949. The theoretical model had a good degree of fit.

The first hypothesis test results (Table 6) supported that visual quality could positively affect safety emotion (*p* < 0.001), but the hypothesis that visual quality could positively affect safety environment perception and control perception was rejected. The second hypothesis test results supported that traffic accessibility positively affected control perception (*p* < 0.001), but the hypothesis that traffic accessibility positively affected safety environment perception and safety emotion was rejected. The third hypothesis test results rejected that facility completeness could positively affect safety environment perception, but the hypothesis that facility completeness could positively affect control perception and safety emotion was accepted. The fourth hypothesis explored the internal mechanism of safety perception, and all hypotheses were supported. Both safety emotion and control perception had a significantly positive impact on safety environment perception, and safety emotion also had a significantly positive impact on control perception.

#### 3.2.3. Mediation Inspection

Intermediary inspection results (Table 7) found that safety emotion played a mediating role between landscape visual quality and safety environment perception (*p* = 0.009), while control perception did not (*p* = 0.145). Both safety emotion and control perception were mediating variables between facility completeness and safety environment perception (*p* = 0.001). Control perception played a mediating role between traffic accessibility and safety environment perception (*p* = 0.007), while safety emotion did not (*p* = 0.587). In the internal mechanism of safety perception, control perception played a moderately mediating role. Control perception was a mediating variable between safety emotion and safety environment perception (*p* = 0.001).

## 4. Discussion

### 4.1. Internal Components of Safety Perception

Used alongside the results of exploratory factor analysis and confirmatory factor analysis in the two research stages (Table 2, Table 4 and Table 5), the compiled safety perception scale had good construct validity, convergent validity, and discriminant validity. The three dimensions of safety perception in an urban forest environment (safety environmental perception, control perception, and safety emotion) could be properly distinguished in people’s cognition and were consistent with the theoretical conception of personal safety perception.

Among the predictors of safety environment perception, in addition to the two physical characteristics of the environment (concealment and entrapment), the empirical prediction of potential threats is also an important basis for judging environmental safety. According to the calculation results (Table 2), the item of worries about dangerous people (0.776) had close factor loading values to the item of worries about animals and insects (0.797). It was common for tourists to worry about animals and insects in the forest. However, we encountered very few dangerous people during our investigation, but dangerous people were still generally perceived by tourists as a potential threat. News reports or past experiences still made them worry about encountering dangerous people in a small space separated by dense trees [39]. Even if the actual management was improved, this insecurity may not be eliminated. In some studies, the experience of danger is often viewed only as a personal characteristic of the tourist [40,41], but when certain threats are widely experienced or feared enough to become widely understood features of the particular environment, they will become an inherent cognitive feature of the environment, even if the feature is not visible. This finding further explains and confirms the correlation between safety perception and threat concern proposed by Farbod [13].

Safety control has always been the focus of safety research. The analysis results of the control perception dimension showed that the control expectation for others had a higher factor load value than the self-judgment of the individual’s control ability, which was the main factor in the control perception dimension. This may be because living creatures have the instinct of self-preservation. When facing danger, people are used to putting themselves in a weak or safe position, hoping that others can face or deal with the danger for them. This subconscious psychological dependence may also explain the findings of Jorgensen et al. [42] that visitors feel safer when they see other users who are engaged in the expected and acceptable use of space because these people are potential objects who may help them. In addition, the research results also validate the findings of Thomas [43] based on a large number of studies on the forest environments and emphasize the important role of movement ease in the perception of danger.

There are few studies at present on safety perception related to positive emotions, and there is only one item on positive emotions (“I feel uncomfortable or comfortable”) in the dimension of safety emotion. However, it can still be proven that the main information reflected by comfort (positive emotion) is consistent with fear, anxiety, and loneliness of the negative emotions in safety cognition. Although positive emotion is more significant in other psychological benefits [44], its influence on safety perception cannot be ignored and requires qualitative study.

### 4.2. Relationship between Urban Forest Service Quality and Safety Perception

Phase 2 of the study confirmed that the internal relationship of safety perception that control perception played a partial mediating role between safety emotion and safety environment perception. Studies on behavioral science suggest that stimulation from threats triggers a physical defense response in the human body, accompanied by the emotion of fear [45]. The results have shown that people can reversely adjust this instinctive response. Tourists with high psychological safety are more inclined to think that their safety has been effectively guaranteed, and this optimistic expectation of safety control can improve their evaluation of environmental safety.

The urban forest service quality measures of visual quality and facility completeness has a direct positive impact on safety emotion and indirectly affects safety environment perception through safety emotion. Good visual quality can counteract the negative emotions of the visitors and bring them peace of mind. In one existing study, a charming natural landscape was also shown to influence tourists’ positive emotions [46]. As a result, safety emotions can also be regarded as the premise that the natural landscape further produces other psychological benefits. Perfect facilities can meet the various recreational needs of tourists in the natural green space, while unsatisfied needs directly trigger their negative emotions, which affects their state of psychological safety. The same interpretation applies to visitors whose primary need is to enjoy the natural landscape of an urban forest. This finding is consistent with the conclusion of other empirical studies that satisfaction of basic needs negatively correlates with negative psychological states (such as anxiety and depression) [47,48]. According to the model analysis findings (Table 6, Figure 4), facility completeness (0.359) has a greater impact on safety emotion than visual quality (0.243). It is normal for tourists to admire the natural scenery while visiting the forest green places. This is their primary objective for visiting the forest. When people enjoy their touring experience, they often overlook facilities. However, when tourists need to rest or find toilets, inadequate or non-existent facilities might leave tourists’ physiological needs unsatisfied. This impact further amplifies insecurity. 

Although visual quality and facility completeness of an urban forest cannot be used as the direct predictor of environmental safety, they can enhance the psychological tolerance of tourists to environmental threats by stabilizing their psychological safety state, thus indirectly affecting their environmental safety assessment. This is consistent with the conclusion of empirical psychological research. Indeed, individuals with low anxiety will show higher tolerance to threats when they are rewarded [49]. In other words, when tourists are immersed in the recreation services of an urban forest, they are more likely to ignore the potential threat of the wild forest itself.

The urban forest service quality measures of accessibility and facility completeness had a direct impact on control perception and indirectly affected safety environment perception through control perception. We noticed that appealing and well-developed sites, such as multi-purpose plazas and rest stops, were often accessed by visitors throughout our survey. The results (Figure 4) showed that in addition to safety facilities and maintenance, the completeness of other facilities is also very important for urban forests, which affects tourists’ judgment of control perception. This is because when faced with danger, people rely on the facilities and tools available around them to fight threats. High-quality facilities can help tourists deal with different emergencies and reduce the probability of danger. The reason that accessibility has a positive impact on tourists’ control perception is that control is a continuous process that lasts until their safety is guaranteed. When tourists are in danger, they will not only control and judge the environment within their visual range but also plan a subsequent evacuation route to ensure that they can quickly return to safe accommodation from the green space or more easily access continuous support and rescue. Tourists’ ambivalence towards nature is largely derived from survival anxiety, and individuals are more likely to realize their vulnerability in the wilderness environment [50]. From the results of the model analysis (Table 6, Figure 4), facility completeness (0.304) can affect control perception more than accessibility (0.138). Tourists tended to pay more attention to the presence and quality of facilities than to accessibility, with signage and monitoring often seen during tours, constantly reminding visitors that they were being protected. The two aspects of accessibility and facility completeness can improve tourists’ subconscious survival confidence by emphasizing social support and lowering tourists’ assessment level of environmental threats. This is consistent with the results of empirical studies, in which the availability of avoidance can keep threats at a low level [51]. When tourists realize that most potential threats can be avoided or solved by the built environment, they will judge wild forests to be non-threatening.

Fieldwork is an extremely effective way to understand tourists’ safety perceptions of urban forests. However, this survey also has limitations. The data collected in this survey do not accurately represent the opinions of people during all months of the year or times of day. Forests vary in their hazard and attractiveness depending on the season. This survey was conducted during the daylight hours of winter and spring when there were numerous tourists. In future, an independent survey is required to investigate periods of low tourism or at night, when the presence of fewer people might exacerbate feelings of insecurity. The study findings’ dependability and utility will be evaluated further following a comparison of various periods. 

The findings of the study also highlight several new research directions. First, the survey of landscape visual quality in this study mainly comes from the evaluation of tourists after viewing the distant wild forest landscape. The aesthetic appreciation will be affected by the sense of distance. Whether the wild landscape preserved at different viewing distances can generate tourists’ aesthetic appreciation and promote their safety emotions is a matter for future research. Second, facility completeness mainly relies on the visual judgment of tourists. It is also meant to explore whether the facilities experience of other sensory forms will affect the perception of safety, such as the comfort of paving and seats and the use of familiar materials. It may subconsciously make tourists feel safe and at ease. 

## 5. Conclusions

This study has discussed the relationship between service quality and safety perception in an urban forest, constructed the theoretical framework of safety perception by evaluating the previous research results on green space safety perception, and compiled a simple safety perception measurement scale. This study evaluated the role of visual quality, facility completeness, and accessibility of urban forest space service quality in the process of safety perception and tested the availability of safety perception scale. The results showed that good service quality in an urban forest green space can improve people’s safety perception, specifically through directly improving tourists’ safety emotion or control perception and then improving their evaluation of environmental safety. It was also found that different service qualities affect the safety perception of an intrinsic mechanism path differently. This reflects the finding that safety perception is a mutual and complicated internal process and also proves that the urban forest environment can improve the safety perception of tourists through targeted service quality optimization. 

## Figures and Tables

**Table 1:** Study site descriptions of Fuzhou Urban Forest Trail.

Research Positions	Location Description	Pavement Material	Common Animals	Distances from Entrances (m)	Number of Monitors
W1	Observation deck with wide view	Glass; wood	Birds, squirrels	1100	1
W2	Wooded horizontal walking trails	Steel frame hollow-out	Snakes, spiders, mosquitoes	800	1
W3	Rest point at the intersection	Steel frame hollow-out	Birds, squirrels, mosquitoes	600	0
W4	High-altitude continuous uphill walking path	Steel frame hollow-out	Birds, snakes	1500	2
W5	Multifunctional event service plaza	Permeable brick	Birds, squirrels, stray dogs	500	3

Information derived from the author’s field records.

**Table 2:** Results of exploratory analysis.

Items	Factor 1	Factor 2	Factor 3
If I am in danger, I can quickly seek help from the managers of the scenic spot	0.854		
If I am in danger, I can easily seek help from others	0.834		
If I am in danger, I can quickly find a shelter to hide or protect myself	0.817		
If I am in danger, I can quickly determine the direction of travel and escape	0.717		
Here, I feel anxious or not anxious		0.875	
Here, I feel alone or not alone		0.822	
Here, I feel uncomfortable or comfortable		0.767	
Here, I feel scared or not scared		0.725	
Here, I am not worried about being infested by annoying or scary animals or insects			0.797
There is no dark or obstructed space around			0.788
Here, I will not worry about encountering dangerous people			0.776
There are no obstacles on the road that prevent me from escaping from danger			0.717
Eigenvalue	6.191	1.536	1.143
Eigenvalue variance explained (%)	25.227	24.912	23.769
Cumulative variance explained (%)	25.227	50.14	73.909

Factor 1, 2, and 3: commonalities of items.

**Table 3:** Reliabilities of categories.

Category	Items	Var.	Mean	S.D.	Alpha
Safety environment perception	There is no dark or obstructed space around	ENV1	4.120	1.061	0.874
	Here, I am not worried about being infested by annoying or scary animals or insects	ENV2	4.160	1.023	
	Here, I will not worry about encountering dangerous people	ENV3	4.260	1.043	
	There are no obstacles on the road that prevent me from escaping from danger	ENV4	4.210	0.992	
Safety emotion	Here, I feel uncomfortable or comfortable	EMO1	4.200	1.055	0.849
	Here, I feel alone or not alone	EMO2	4.440	0.868	
	Here, I feel anxious or not anxious	EMO3	4.500	0.820	
	Here, I feel scared or not scared	EMO4	4.320	0.882	
Control perception	If I am in danger, I can quickly seek help from the managers of the scenic spot	CON1	4.260	0.924	0.854
	If I am in danger, I can easily seek help from others	CON2	3.790	1.126	
	If I am in danger, I can quickly find a shelter to hide or protect myself	CON3	3.800	1.127	
	If I am in danger, I can quickly determine the direction of travel and escape	CON4	3.680	1.108	
Visual quality	The ecological woodland here is very ornamental	VIS1	4.280	0.871	0.886
	The landscape here is very colorful	VIS2	4.150	0.945	
	Here undulating terrain of the mountain is very beautiful	VIS3	4.350	0.825	
	The design of the trails here is very beautiful	VIS4	4.410	0.828	
Accessibility	It is convenient to get here by public transportation in the city	ACC1	3.680	0.840	0.749
	The time it took to get here was in line with my expectations	ACC2	3.440	0.898	
	The number and location of entrances and exits are convenient for me to reach	ACC3	3.560	0.957	
Facility completeness	The identification systems here are very complete and numerous	FAC1	4.220	0.885	0.811
	The monitoring facilities and protective fences here are very complete	FAC2	4.250	0.856	
	The sanitation facilities here are very complete	FAC3	4.370	0.832	
	The rest facilities here are very complete	FAC4	3.990	1.028	

S.D., Standard deviation.

**Table 4:** Convergent validity.

Factor	Variables	Std. Coefficient	AVE	CR
Safety environment perception	ESP1	0.721	0.640	0.876
	ESP2	0.795		
	ESP3	0.88		
	ESP4	0.797		
Safety emotion	EMO1	0.748	0.604	0.858
	EMO2	0.81		
	EMO3	0.866		
	EMO4	0.671		
Control perception	BEH1	0.866	0.599	0.854
	BEH2	0.857		
	BEH3	0.754		
	BEH4	0.583		
Visual quality	AES1	0.87	0.668	0.889
	AES2	0.868		
	AES3	0.804		
	AES4	0.717		
Accessibility	ACC1	0.761	0.507	0.755
	ACC2	0.715		
	ACC3	0.656		
Facility completeness	FAC1	0.837	0.552	0.829
	FAC2	0.817		
	FAC3	0.725		
	FAC4	0.561		

Notes: CR, construct reliability; AVE, average variance extracted.

**Table 5:** Discriminant validity and the correlations of variables.

Variable	Safety Environment Perception	Safety Emotion	Control Perception	Visual Quality	Traffic Accessibility	Facility Completeness
Safety environment perception	0.640					
Safety emotion	0.762 ***	0.604				
Control perception	0.560 ***	0.521 ***	0.599			
Visual quality	0.472 ***	0.518 ***	0.498 ***	0.668		
Accessibility	0.192 ***	0.191 ***	0.312 ***	0.197 ***	0.507	
Facility completeness	0.499 ***	0.532 ***	0.574 ***	0.697 ***	0.328 ***	0.552
Square root of AVE	0.800	0.777	0.774	0.817	0.712	0.743

*** indicates that the *p* value is less than 0.001, and the diagonal line is the amount of AVE evaluation variance variation extraction.

**Table 6:** Summary of hypotheses’ results.

Hypothesis	Estimate	S.E.	C.R.	P	Result
H1-1. Visual quality → safety environment perception	0.018	0.077	0.329	0.742	Not accepted
H1-2. Visual quality → control perception	0.114	0.063	1.773	0.076	Not accepted
H1-3. Visual quality → safety emotion	0.243	0.084	3.517	0.000	Accepted
H2-1. Accessibility → safety environment perception	−0.008	0.054	−0.181	0.857	Not accepted
H2-2. Accessibility → control perception	0.138	0.044	2.724	0.006	Accepted
H2-3. Accessibility → safety emotion	0.028	0.056	0.523	0.601	Not accepted
H3-1. Facility completeness → safety environment perception	0.038	0.086	0.589	0.556	Not accepted
H3-2. Facility completeness → control perception	0.304	0.072	3.966	0.000	Accepted
H3-3. Facility completeness → safety emotion	0.359	0.091	4.53	0.000	Accepted
H4-1. Safety emotion → safety environment perception	0.627	0.069	10.427	0.000	Accepted
H4-2. Control perception → safety environment perception	0.204	0.076	3.792	0.000	Accepted
H4-3. Safety emotion → control perception	0.277	0.047	4.76	0.000	Accepted

S.E.: Approximate standard error; C.R.: Critical ratio.

**Table 7:** Test of bootstrap indirect effect.

Parameter	Estimate	SE	Bias-Corrected 95%CI		
			Lower	Upper	P
VIS → CON → ENV	0.032	0.027	−0.014	0.095	0.146
VIS → EMO → ENV	0.212	0.09	0.058	0.407	0.009
FAC → CON → ENV	0.082	0.038	0.025	0.18	0.001
FAC → EMO → ENV	0.299	0.09	0.144	0.494	0.001
ACC → CON → ENV	0.034	0.018	0.007	0.084	0.007
ACC → EMO → ENV	0.021	0.047	−0.069	0.118	0.583
EMO → CON → ENV	0.065	0.029	0.022	0.133	0.001

Note: SE, bootstrap standard error; CI, confidence interval; VIS, visual quality; ACC, accessibility; FAC, facility completeness; ENV, safety environment perception; EMO, safety emotion; CON, control perception.

## Data Availability

The data used to support the findings of this study are available from the corresponding author upon request.
